# The Role of Transforming Growth Factor Beta-1 in the Progression of HIV/AIDS and Development of Non-AIDS-Defining Fibrotic Disorders

**DOI:** 10.3389/fimmu.2017.01461

**Published:** 2017-11-02

**Authors:** Annette J. Theron, Ronald Anderson, Theresa M. Rossouw, Helen C. Steel

**Affiliations:** ^1^Faculty of Health Sciences, Department of Immunology, Institute for Cellular and Molecular Medicine, University of Pretoria, Pretoria, South Africa; ^2^Tshwane Academic Division of the National Health Laboratory Service, Pretoria, South Africa

**Keywords:** highly active antiretroviral therapy, immunosuppression, lymphoid fibrosis, macrophages, non-AIDS-defining disorders, organ fibrosis, T regulatory cells, transforming growth factor-beta

## Abstract

Even after attainment of sustained viral suppression following implementation of highly active antiretroviral therapy, HIV-infected persons continue to experience persistent, low-grade, systemic inflammation. Among other mechanisms, this appears to result from ongoing microbial translocation from a damaged gastrointestinal tract. This HIV-related chronic inflammatory response is paralleled by counteracting, but only partially effective, biological anti-inflammatory processes. Paradoxically, however, this anti-inflammatory response not only exacerbates immunosuppression but also predisposes for development of non-AIDS-related, non-communicable disorders. With respect to the pathogenesis of both sustained immunosuppression and the increased frequency of non-AIDS-related disorders, the anti-inflammatory/profibrotic cytokine, transforming growth factor-β1 (TGF-β1), which remains persistently elevated in both untreated and virally suppressed HIV-infected persons, may provide a common link. In this context, the current review is focused on two different, albeit related, harmful activities of TGF-β1 in HIV infection. First, on the spectrum of anti-inflammatory/immunosuppressive activities of TGF-β1 and the involvement of this cytokine, derived predominantly from T regulatory cells, in driving disease progression in HIV-infected persons *via* both non-fibrotic and profibrotic mechanisms. Second, the possible involvement of sustained elevations in circulating and tissue TGF-β1 in the pathogenesis of non-AIDS-defining cardiovascular, hepatic, pulmonary and renal disorders, together with a brief comment on potential TGF-β1-targeted therapeutic strategies.

## Introduction

It is well established that the direct cytopathic effects of HIV, as well as destruction by cytotoxic CD8^+^ T cells, are the major contributors to chronic depletion of CD4^+^ T cells, both in the circulation and secondary lymphoid tissues, resulting in the progressive immunosuppression which culminates in the development of AIDS (1, 2). However, it is also now well recognized that HIV infection-related immunosuppression is exacerbated by several other, albeit indirect mechanisms, which may even precede significant loss of CD4^+^ T cells. Most noteworthy among these is secondary immunosuppression resulting from the sustained activation of the anti-inflammatory machinery of the host, mobilized to counteract not only HIV-mediated chronic immune activation but also chronic inflammation due to microbial translocation. Mechanistically, break down of the tight epithelial barrier of the gastrointestinal tract (GIT) results from a combination of factors, namely preferential infection and depletion of CCR5-expressing CD4^+^ T lymphocytes, accumulation of inflammatory cells and a concomitant decrease in cells that regulate epithelial homeostasis, alterations in the regulation of tight junction protein expression, and possibly epithelial and enterocyte apoptosis (3–5). This allows proinflammatory microbial products to translocate into the lamina propria of the GIT, and, eventually, into the systemic circulation (5).

With respect to HIV-related secondary immunosuppression, a number of studies has implicated chronic over-production of the anti-inflammatory, profibrotic cytokine, transforming growth factor beta-1 (TGF-β1), as a major cause of immunosuppression in HIV infection (6, 7). Not only does this cytokine promote immunosuppression directly by targeting cells of both the adaptive and innate immune systems (8, 9), but also indirectly *via* its profibrotic activity, resulting in the depletion of T cells in secondary lymphoid tissues, while also compromising repopulation of T cell-dependent zones following implementation of antiretroviral therapy (10).

Following brief descriptions of the subtypes of TGF-β, cellular origins and the intracellular signaling mechanisms utilized by this cytokine, as well as an overview of its spectrum of immunosuppressive activities, this review is focused primarily on the role of TGF-β, specifically TGF-β1, in driving HIV-associated, chronic, secondary immunosuppression. Particular emphasis is placed on: (i) T regulatory cells (Tregs) as a major source of the cytokine; (ii) profibrotic destruction of the architecture of secondary lymphoid organs/tissues; (iii) pro-oxidative interactions with macrophages, all three of which drive disease progression; and (iv) the involvement of sustained elevations of TGF-β1 in the pathogenesis of profibrotic, HIV-associated, non-AIDS-defining conditions, specifically those of cardiovascular, hepatic, pulmonary, and renal origin, as well as the possible role of highly active antiretroviral therapy (HAART).

## Transforming Growth Factor Beta

Transforming growth factor beta belongs to a superfamily of cytokines that includes three TGF-β isoforms, as well as activins, bone morphogenetic proteins, growth and differentiation factors, and others (11). According to Blobe et al., “virtually every cell in the body, including epithelial, endothelial, hematopoietic, neuronal, and connective-tissue cells, produces TGF-β and has receptors for it” (12), while Prud’homme and Piccirillo state that TGF-βs are involved in a variety of different biological processes including embryonic development, fibrosis, wound healing, angiogenesis, hematopoesis, and regulation of the immune response (13).

All three isoforms (β1, β2, and β3) are produced by mammals with TGF-β1 being most common in the immune system, in which it functions as an important pleiotropic cytokine with potent immunoregulatory properties (14), the importance of which is underscored by observations that mice lacking TGF-β1 die of multiorgan inflammation early in life (15, 16).

The biological activities of TGF-β are achieved *via* binding of the cytokine to type II receptors (TGF-βRII) on target cells. This, in turn, causes activation of type 1 receptors (TGF-βRI), resulting in the phosphorylation and activation of the Sma- and Mad-related protein (Smad) transcription factors, Smad 2 and Smad3 (17). Phosphorylated receptor-regulated Smads then bind to the co-Smad, Smad 4, which translocates to the nucleus to modulate gene expression (17). TGF-β isoforms may also exert their effects through Smad-independent pathways (17, 18). Importantly, TGF-β is synthesized in an inactive form and must be converted to its active form prior to binding to TGF-βRII (19). Active TGF-β is produced by proteolytic processes that promote the dissociation of inactive TGF-β from latency-associated protein or latent-TGF-β binding protein, which maintains TGF-β in an inactive state (20).

Transforming growth factor beta-1 is encoded by a distinct gene located in the 19q13.2 chromosomal region, which comprises seven exons separated by six very large introns (21). Functional genetic variations of the TGF-β1 gene (gene polymorphisms) have been linked to variations of protein expression or functionality (22). According to Martelossi Cebinelli et al., eight single-nucleotide polymorphisms (SNPs) (rs2317130, rs11466313, rs1800468, rs1800469, rs11466314, rs1800471, rs1800470, and rs11466316) as well as one deletion/insertion polymorphism, have been shown to affect TGF-β1 expression (21). The authors state that these SNPs interfere with either transcriptional regulation of TGF-β1 or its synthesis (21). An earlier study by Kruit et al. alluded to the fact that TGF-β1 gene SNPs, which are present in codon 10 (Leu10Pro/rs1800470) and codon 25 (Arg25Pro/rs1800471), can contribute to variations in TGF-β1 production (22). The bi-allelic polymorphism changes the amino acid at codon 10 from leucine to proline, or at codon 25, from arginine to proline, which is associated with lower synthesis of TGF-β1 (23). The TGF-β1 high producer genotype may be a risk factor for certain conditions, e.g., those associated with fibrosis (23).

## Cellular Targets of the Immunosuppressive Activities of TGF-β1

### Effects on Cells of the Adaptive Immune System

Transforming growth factor beta-1 utilizes various mechanisms to mediate suppression of the reactivity of T lymphocytes (8, 14, 20). In this context, the cytokine is a potent inhibitor of both T-helper (Th)1 and Th2 cell differentiation and proliferation *in vitro*, which is achieved *via* inhibition of production of the transcription factors, T bet, and GATA-3, respectively (24, 25). These mechanisms are likely to account for the suppressive effects of TGF-β1 on production of the cytokines interferon (IFN)-γ, interleukin (IL)-2, and IL-4 (14), and possibly the rapid loss of the β2 subunit of the IL-12 receptor (IL-12R) on CD4^+^ T cells, which then become unresponsive to IL-12 (26). In addition to these activities, TGF-β1 has also been reported to induce apoptosis of CD4^+^ T lymphocytes following macrophage-tropic (R5) HIV-1 infection, by reducing levels of Bcl-2 (survival signal), as well as by increasing those of the intracellular death signals, caspase-3, apoptosis-inducing factor, and BH3 interacting-domain death agonist (27).

Notwithstanding its suppressive effects on CD4^+^ T cells, TGF-β1 also regulates the proliferative and effector functions of CD8^+^ T cells. In the case of the latter, the synthesis of IFN-γ, the exocytic release of perforins and granzymes, and the expression of Fas ligand, which collectively contribute to the cytotoxicity of CD8^+^ T cells, are all downregulated by TGF-β1 (28, 29).

Transforming growth factor beta-1 also inhibits immune responses indirectly *via* regulation of CD4^+^, CD25^+^, Foxp3^+^ Tregs that potently suppress T cell functions. Contrary to its aforementioned proapoptotic effects on CD4^+^ T helper cells, TGF-β1 in this setting has been reported to protect Tregs from apoptosis during thymic development (30), as well as promoting the differentiation of induced Tregs. With regard to the latter, studies have shown that TGF-β1 is critically involved in mediating the transition of naive, peripheral CD4^+^, CD25^−^ non-Tregs into functionally mature CD4^+^, CD25^+^, Foxp3^+^ Tregs (31, 32).

Transforming growth factor beta-1 also regulates humoral immune responses, with a very recent study having reported that both TGF-β1 and TGF-β3 suppress the survival and proliferation of B cells, as well as their differentiation into antibody-secreting cells (9).

### Effects on Cells of the Innate Immune System

Transforming growth factor beta-1 also regulates the reactivities of cells of the innate immune system. In this context, the cytokine was shown to inhibit the production of IFN-γ by human natural killer (NK) cells, as well as antibody-dependent cellular cytotoxicity (ADCC) induced by CD16 activation. The effect on ADCC was attributed to TGF-β1-mediated inhibition of synthesis of granzyme A and granzyme B protein, which was associated with decreased mRNA expression (33). TGF-β1 was also shown to decrease the expression of the activating receptors, natural killer protein 30 (NKp30), natural killer group 2D (NKG2D), and DNAX accessory molecule-1 (DNAM-1), leading to reduced synthesis and release of IFN-γ, as well as granule exocytosis, resulting in attenuation of tumor killing (34). A more recent study demonstrated that TGF-β1 inhibited the activation and functions of NK cells by repressing the mammalian target of rapamycin (mTOR) pathway, a central regulator of cellular metabolism and cytotoxic functions of these cells (35).

A number of studies [reviewed in Ref. (36–38)] have reported that TGF-β1 also has various regulatory effects on dendritic cells (DCs). In an earlier study, this cytokine was shown to inhibit the *in vitro* activation and maturation of DCs (39). In addition to inducing DC apoptosis (40), TGF-β1 has also been reported to impede critical functions of these cells, including migration (41), expression of costimulatory (CD40, CD80, and CD86) and HLA class II molecules (37, 38), antigen-presenting capacity (36), and production of tumor necrosis factor-α, IFN-α, and IL-12 (38).

The proinflammatory functions of macrophages are also negatively affected by TGF-β1. These include inhibition of expression of inducible nitric oxide synthase (iNOS) and matrix metalloproteinase (MMP)-12 (42). In addition, MyD88-dependent Toll-like receptor (TLR)-activated signaling pathways are also downregulated by this cytokine (43). In the case of neutrophils, an earlier study reported that TGF-β1 is a potent chemotactic and activating factor for these cells (44). In contrast, others have reported that TGF-β1 inhibits neutrophil transmigration through activated endothelium by down-regulating the expression of endothelial E-selectin and IL-8 (45), as well as suppressing neutrophil degranulation (46).

These immunosuppressive effects of TGF-β1 are summarized in Table 1.

**Table 1:** Summary of the immunosuppressive effects of TGF-β1.

Adaptive immunity		

Cell type	Effects	Reference
T lymphocytes	Blocks Th1 and Th2 differentiation	(24, 25)
	Suppresses production of IFNγ, IL-2, IL-4	(14)
	Promotes loss of IL-12 receptor β subunit	(26)
	Causes apoptosis of CD4^+^ lymphocytes	(27)
CD8^+^ T lymphocytes	Downregulates synthesis/expression of perforins, granzymes, Fas ligand, IFNγ	(28)
T regulatory cells (Tregs)	Protects Tregs from apoptosis during thymic development	(30)
	Promotes differentiation of induced Tregs	(31, 32)
B lymphocytes	Suppresses survival and proliferation of B cells	(9)
	Suppresses differentiation into antibody secreting cells and antibody production	(9)

**Innate immunity**		

Natural killer cells	Inhibits antibody-dependent cellular cytotoxicity	(33)
	Decreases expression of activation markers NKp30, NKG2D, DNAM-1, leading to reduced release of IFNγ, granule exocytosis, and tumor killing	(34)
	Inhibits activation and functions by suppressing the mTOR pathway	(35)
Dendritic cells	Inhibits activation and maturation of DCs	(39)
	Induces apoptosis of DCs	(40)
	Interferes with migration	(41)
	Downregulates expression of costimulatory molecules and HLA II molecules	(37, 38)
	Reduces antigen presenting capacity	(36)
	Inhibits production of TNF, IFNα, IL-12	(38)
Macrophages	Inhibits induction of iNOS, MMP-12	(42)
	Suppresses MyD88-dependent TLR signaling pathway	(43)
Neutrophils	Inhibits neutrophil transmigration across vascular endothelium	(45)
	Suppresses neutrophil degranulation	(46)

*mTOR, mammalian target of rapamycin; NKp30, natural killer protein 30; NKG2D, natural killer group 2D; DNAM-1, DNAX accessory molecule-1; DC, dendritic cells; iNOS, inducible nitric oxide synthase; MMP-12, matrix metalloproteinase-12; MyD88, myeloid differentiation protein 88*.

## Role of TGF-β1 in HIV Infection-Associated Immunosuppression

### Increased Blood and Tissue Levels of TGF-β1 in HIV Infection

Possibly the first article documenting increases of plasma/serum TGF-β in treatment naive male patients with AIDS was published by Allen et al. (47). Several later reports also documented increased concentrations of this cytokine in the blood, lymphoid tissues and cerebrospinal fluid of HIV-infected individuals (6, 48–53). In addition, two other earlier studies reported that isolated peripheral blood mononuclear cells (PBMCs) from HIV-infected persons spontaneously released high levels of TGF-β, which was associated with selective upregulation of the TGF-β1 isoform (54, 55). In this setting, increased levels of this cytokine were associated with defective T cell proliferation to recall antigens, as well as B lymphocyte proliferative responses and immunoglobulin production, possibly related to the proapoptotic mechanisms described above (27), all of which were restored by the addition of a TGF-β1-neutralizing monoclonal antibody (54). Important caveats to bear in mind when measuring TGF-β1 in biological matrices are the requirement for stringent control of activation procedures, such as acidification, which precede measurement of this cytokine, as well as careful processing of blood specimens to avoid contamination with platelet-derived TGF-β1.

HIV-1 proteins may also contribute to the production of TGF-β1. The HIV-1 transactivator of transcription (Tat) protein has been shown to induce synthesis of TGF-β1 by human leukocytes (56), which may underpin the immunosuppressive effects of Tat (56). It has also been demonstrated that HIV-1 glycoprotein (gp)160 induces significant TGF-β mRNA expression and TGF-β1 secretion in PBMC from HIV-seronegative, healthy donors (57), which resulted from the CD4–gp160 interaction. In addition, Garba et al. demonstrated that HIV antigens induced TGF-β1 secretion by CD8^+^ T cells, which resulted in inhibition of production of IFN-β in response to HIV, as well as unrelated antigens (58).

### Involvement of Regulatory T Cells in TGF-β1-Mediated Immunosuppression in HIV Infection

The numbers of Tregs are highly increased in the mucosa and lymphoid tissues of untreated HIV patients and are associated with disease progression (49, 59, 60). Tregs exert their suppressive functions through various mechanisms including the production of TGF-β1, as well as another broadly -active anti-inflammatory cytokine, *viz*. IL-10 (61). TGF-β1 is normally a protective, anti-inflammatory cytokine, but its overproduction may have serious pathogenic effects. As mentioned above, increases in the concentrations of TGF-β1 in the circulation of HIV-1 infected patients have been reported in several studies, and, in one of these, were found to be highly correlated with increased numbers of circulating Tregs (*r* = 0.921, *p* = 0.001) (52), indicating that the cytokine is likely to originate predominantly from Tregs. This contention is supported by findings that elevated numbers of circulating Tregs and concentrations of TGF-β1 correlate with increased levels of circulating biomarkers of chronic immune activation (monocyte- and lymphocyte-derived activation markers) and bacterial translocation (51–53). As mentioned earlier, these are considered to be major drivers of immunosuppression and progression to AIDS (3–5, 62). This ongoing counterbalancing activity of Tregs and associated cytokines, which paradoxically also contributes to progressive immunosuppression, may underpin the prolonged asymptomatic phase of HIV infection. Worryingly, however, several studies have reported that even in the face of administration of virally suppressive HAART for periods of 6–12 months, circulating levels of both Tregs and TGF-β1 remained elevated. This appears to be consistent with ongoing chronic immune activation and its associated risks of persistent, albeit of lesser magnitude, immunosuppression, and development of HIV-associated, non-AIDS-defining conditions (6, 7, 51–53).

In this context, it is noteworthy that Chevalier and Weiss have proposed that Treg-mediated dampening of chronic immune activation may be less effective in untreated HIV-infected patients with high levels of immune activation as found in primary and chronic infection, compared with HAART-treated patients who have low levels of residual immune activation (63). These authors also proposed that HIV-driven Treg expansion may occur not only as a result of chronic immune activation, but may also be a consequence of both increased survival and enhanced production of these cells in the thymus (63). This contention is supported by the findings of two earlier studies. First, Chen et al., as alluded to above, reported that TGF-β1, as well as being produced by Tregs, also caused the induction of these cells by promoting the transition of CD4^+^, CD25^−^ cells to CD4^+^, CD25^+^ Tregs that expressed the *Foxp3* gene (31). These cells produced TGF-β1, but not Th1 or Th2 cytokines, and effectively suppressed T cell proliferation *in vitro* (31). Second, Ji and Cloyd have demonstrated that CD4^+^, CD25^+^ Treg cell binding to HIV-1 prolongs survival and enhances both the suppressive functions and accumulation of these cells in peripheral lymph nodes and mucosal lymphoid tissues (64).

## TGF-β1-Mediated Lymphoid Tissue Fibrosis as a Contributor to HIV Infection-Associated Immunosuppression

According to Deeks, it remains an unanswered question as to why CD4^+^ T cells destroyed during HIV infection are not replaced by the immune system as only a “small fraction of cells are infected and killed on a daily basis after the acute phase of the disease” (65). Moreover, a substantial number of HIV-infected patients do not experience significant increases in their peripheral blood CD4 counts after initiation of HAART despite low HIV viral loads (2). These anomalies may be explained in part by the “damaged lymphoid tissue hypothesis” in which TGF-β1 plays a major role as a profibrotic agent in disrupting the fibroblastic reticular cell (FRC) network of secondary lymphoid structures.

The FRC network is located in the T cell zone of lymphoid tissues and provides a mechanical infrastructure that enables lymphocyte recruitment and organization, as well as promoting contact between T cells and antigen-presenting DCs. FRC-derived chemokines and cytokines, particularly IL-7, are critically involved in maintaining naive T cell recruitment and survival within lymphoid tissue (66). It has been demonstrated, however, that in HIV infection, as well as in simian immunodeficiency virus (SIV) infection of rhesus macaques, lymphoid tissue undergoes collagen deposition and fibrosis (2, 10), leading to the loss of the FRC network and reduced availability of IL-7 (2, 10). Consequently, there is an increased tendency for naive CD4^+^ and CD8^+^ T cell populations to undergo apoptosis, leading to a progressive reduction in the numbers of these cells (2). Depletion of T cells also leads to loss of lymphotoxin β, on which maintenance of the FRC depends, thereby exacerbating failure of production of IL-7, resulting in a vicious cycle of T cell and FRC depletion (10). Reversal of these effects was only found to be optimal when HAART was initiated in the acute stage of the infection (2).

These same authors also detected an increase in Tregs positive for TGF-β1, as well as an increase in the numbers of fibroblasts expressing the TGF-β1RII in lymphoid tissue, favoring chronic activation of the TGF-β1 signaling pathway, with consequent augmentation of production of procollagen by fibroblasts. Moreover, TGF-β1 also enhanced the expression of chitinase 3-like-1 activity in fibroblasts, thereby contributing to procollagen maturation and formation of collagen fibrils in the lymphoid tissue of HIV-infected patients (10). Taken together, these events strongly implicate potent and sustained Treg responses with production of profibrogenic “TGF-β which, in turn, causes collagen deposition, tissue fibrosis, the loss of the FRC network” and depletion of naive T cells (65).

**Figure 1:**
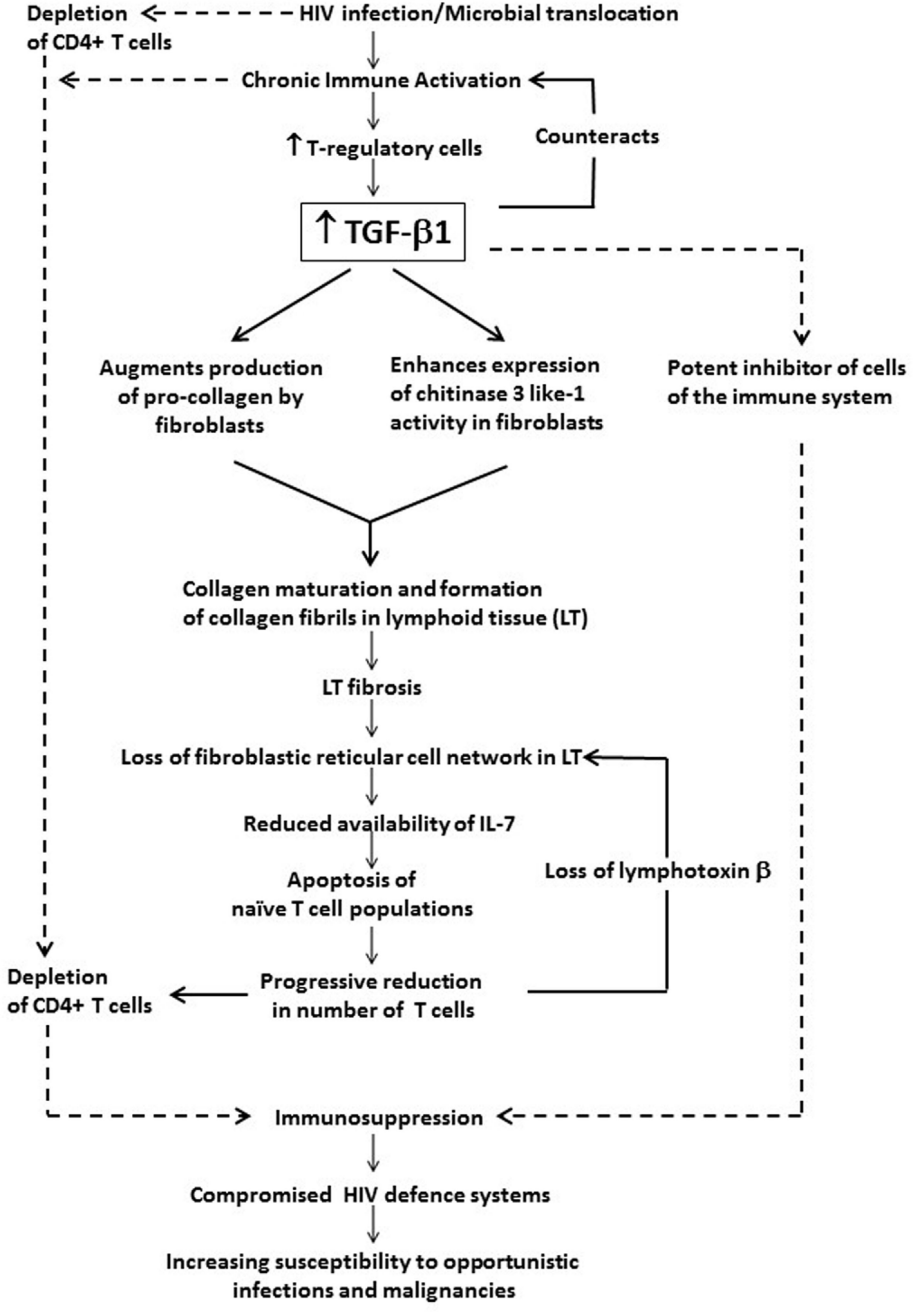
The potential contribution of chronic systemic immune activation in HIV infection to driving sustained increases in circulating and tissue concentrations of transforming growth factor beta-1 (TGF-β1). This cytokine, which appears to originate predominantly from T regulatory cells (Tregs), exacerbates immunosuppression and disease progression not only *via* its direct inhibitory effects on CD4^+^ T cells and other cells of the adaptive immune system, but also *via* prevention of T cell repopulation of secondary lymphoid tissue *via* profibrotic activity.

The association of chronic systemic immune activation in HIV infection with persistently increased levels of TGF-β1 and the role of this cytokine in immunosuppression and disease progression are summarized in Figure 1.

## Oxidative Stress as a Cause of Production of TGF-β1 by Macrophages

In addition to the immunosuppressive effects on immune cells, cytokines of the TGF-β family have also been reported to increase the production of reactive oxygen species (ROS) by various cell types, resulting in decreased concentrations of intracellular glutathione (GSH), the most abundant intracellular free thiol and an important antioxidant (67). ROS, in turn, augment the production and reactivity of TGF-β cytokines *via* induction of gene expression and activation of latent TGF-β (67).

In this context, it is noteworthy that monocyte-derived macrophages derived from monocytes isolated from the blood of HIV-infected individuals have been reported to have decreased ratios of GSH:oxidized glutathione (GSSG), consistent with increased intracellular oxidative stress (50). This contention was supported by several findings, including decreased activation of genes encoding enzymes involved in the synthesis of GSH, increased levels of lipid peroxidation products in macrophage lysates, and increased concentrations of the cytokines IL-1, IL-17, and TGF-β1 in cell lysates and plasma (50). In a follow-up study, administration of liposomal glutathione supplements (L-GSH) to HIV-infected individuals was found to alleviate oxidative stress and correct the Th1 cytokine imbalance, while decreasing the levels of TGF-β1 and IL-10 (68).

Although unproven, microbial translocation in HIV infection resulting in persistent macrophage activation, possibly due to chronic stimulation of TLRs by microbial products, may also drive the production of TGF-β1 by these cells, contributing to sustained immunosuppression and predisposition to development of HIV-associated, non-AIDS-defining conditions (69, 70).

## TGF-β1 and Disease Progression in HIV Infection

Wiercińska-Drapalo et al. investigated the levels of circulating TGF-β1 and possible relationships with clinical parameters of HIV infection in 66 patients at different stages of HIV infection, 35 of whom were receiving HAART. A near twofold increase in plasma TGF-β1 was observed in HIV-infected patients compared to 20 healthy controls (6). Patients with a CD4 count below 200 cells/μL demonstrated the highest levels of TGF-β1, which were significantly and inversely correlated with circulating CD4 and CD8 counts, while no significant correlation was found with HIV viral load. Furthermore, patients in the symptomatic phase of the disease showed increased levels of TGF-β1 compared to those in the asymptomatic phase. No relationship was found between use of HAART and levels of TGF-β1. The authors proposed that TGF-β1 was associated with disease progression in HIV infection (6).

In a more recent study, TGF-β1 levels, as well as those of Th1 and Th2 cytokines, were also measured in HIV-infected patients stratified according to disease severity, i.e., non-progressive and progressive HIV infection (7). The latter group received HAART. The authors observed that TGF-β1 levels were increased in all HIV-infected patients, but even more so in patients with progressive disease when compared to those with non-progressive disease and healthy controls. These authors also observed that plasma levels of TGF-β1 and IL-10 in patients with non-progressive infection correlated inversely with CD4^+^ T cell counts and CD4:CD8 ratios, and proposed that these increases in TGF-β1 and IL-10 promote an immunosuppressive environment conducive to disease progression (7). In contrast, however, a study by Gaardbo et al. reported that Treg percentages and levels of TGF-β1 were comparable in healthy controls, HIV-infected viraemic controllers, elite controllers, long-term non-progressors, and progressors (71). The similar percentages of Tregs (and levels of TGF-β1) could possibly be attributed to the fact that in this study all patients had normal CD4^+^ T cell counts, as evidence suggests that increased Treg percentages are preferentially found in patients with low CD4^+^ T cell counts (71).

Although persistently elevated levels of circulating and tissue TGF-β1 have been implicated in sustained immunosuppression and progression to AIDS, it is also possible, albeit speculative, that this mechanism may also contribute to the pathogenesis of non-AIDS-related disorders, which is the topic of the remaining sections of this review.

## Changing Patterns of Mortality in HIV/AIDS

The introduction of HAART in 1996, while resulting in significantly decreased mortality due to AIDS-related causes, has also been accompanied by an awareness of the emergence of a changing pattern of HIV/AIDS-related mortality. This is attributable to an increased prevalence of non-AIDS-defining malignancies and other non-communicable diseases (NCDs), particularly those of cardiovascular, hepatic, and renal origin (72–74). With the passage of time, this trend has become even more apparent (75–81), particularly in settings of advanced health care, most evident in developed countries, where mortality from HIV-related, non-AIDS-defining conditions may even exceed mortality due to opportunistic infections (82, 83). Among developed countries, however, the United Kingdom is an exception, where despite the ready accessibility of HAART, AIDS continues to account for the majority of deaths in HIV-infected persons (84). Persons at highest risk for development of HIV-related, non-AIDS-defining NCDs appear to be those who experience chronic immune activation associated with sustained, intense immunosuppression, even in the face of HAART-mediated viral suppression (75, 77–79).

An increasing body of evidence has implicated ongoing microbial translocation-associated chronic activation of monocytes/macrophages as a major contributor to the persistent systemic inflammation which underpins the pathogenesis of serious non-AIDS-defining morbidities and associated mortality (85–93). This contention is based on the frequency of reports that have consistently noted significant associations between non-AIDS-defining morbidities and/or mortality with elevated levels of circulating biomarkers of microbial translocation. These include intestinal fatty acid-binding protein and bacterial endotoxin (85, 86), as well as biomarkers indicative of monocyte/macrophage activation, particularly soluble CD14 (sCD14), IL-6 (85–91), and the macrophage scavenger receptor, sCD163, considered to be a more selective biomarker of macrophage activation and proliferation (91–93).

In addition, it is noteworthy that persistent systemic inflammation may also underpin the profibrotic mechanisms that have been implicated in the pathogenesis of HIV-related cardiovascular, hepatic, pulmonary, and renal disease (94–97). Surprisingly, however, with the notable exceptions of two earlier publications focused on HIV-related renal disease (98, 99), the involvement of persistently elevated levels of TGF-β1, in the pathogenesis of these non-AIDS-defining disorders remains largely unexplored. The remaining sections of this review are focused on the role of profibrotic mechanisms in the pathogenesis of HIV-related cardiovascular, renal, hepatic, and pulmonary disorders, and, by implication, the involvement of TGF-β1.

## Cardiovascular Disease (CVD) and Its Relationship to Haart-Mediated Cardiotoxicity and Inflammation-Associated Cardiac Fibrosis in HIV Infection

It is now well recognized that people living with HIV in the HAART era have a significantly increased risk for development of various types of CVD. These include coronary artery disease with its associated risk of types 1 and 2 myocardial infarction (MI), heart failure, arrhythmias, sudden cardiac death, cerebrovascular disease, pericardial diseases, and pulmonary hypertension (100–107). Several mechanisms have been proposed to contribute to the pathogenesis of CVD in HIV infection. Notwithstanding HIV-associated myocarditis and the existence of established risk factors such as older age, smoking and preexisting diabetes, hypertension, dyslipidaemia and renal dysfunction, as well as concomitant hepatitis C virus (HCV) infection (108), the most prominent contributors to CVD in HIV-infected patients are believed to be the adverse effects of certain components of HAART on the vasculature and heart, and, in particular, ongoing chronic immune activation encompassing both HAART-naive patients and even those who have achieved a significant degree of viral suppression (109).

### HAART-Associated Cardiotoxicity

An earlier study reported that exposure of rat ventricular myocytes to therapeutically relevant concentrations of the nucleoside reverse transcriptase inhibitor (NRTI), zidovudine, *in vitro*, resulted in damage to mitochondria (110). In the same study, exposure of human primary coronary artery endothelial cells and brain microvascular endothelial cells to zidovudine, or the protease inhibitor, indinavir, or, albeit to a lesser extent, the non-NTRI, efavirenz, caused disruption of intercellular gaps, as well as decreased transendothelial electrical resistance (110). In a subsequent study, administration of the NRTIs, zidovudine, and stavudine, but not lamivudine, to transgenic mice expressing the human mitochondrial deoxynucleotide carrier targeted to murine myocardium, resulted in increased left ventricular mass, damage to mitochondrial DNA and mitochondrial destruction (111). Other HAART-related mechanisms which predispose to development of CVD, particularly in the case of HIV protease inhibitors, include “elevations in serum levels of total cholesterol and triglycerides, decreased high-density lipoprotein cholesterol, lipodystrophy, insulin resistance and diabetes” reminiscent of the metabolic syndrome (112–114).

Protease inhibitors have also been proposed to promote myocardial oxidative stress, interfering, among other targets, with ion channel function (115). In the context of the current review, it is noteworthy that the protease inhibitor, ritonavir, has been implicated in the pathogenesis of HIV infection-associated myocardial fibrosis by TGF-β1-dependent mechanisms (116). These involve either direct activation of platelets resulting in the release of stored TGF-β1 by these cells, and/or by potentiation of production of TGF-β1 by other cell types *via* attenuation of the intracellular proteolysis of the signal-transducing adaptor protein, TNF receptor-associated factor 6 (116), which, in turn, is required for SMAD-independent synthesis of the cytokine (117). With respect to the possible involvement of these TGF-β1-associated mechanisms in the pathogenesis of HIV-related myocardial fibrosis, it is noteworthy that systemic activation of platelets has been described in both treatment-naive HIV-infected patients (*n* = 35) (118), as well as in patients on virally suppressive HAART (*n* = 73), which included a protease inhibitor in 76.7% of cases (119). However, possible associations between elevations in the concentrations of systemic biomarkers of platelet activation and types of antiretroviral agent were not mentioned in the latter study (119).

A systematic review and meta-analysis of eight studies, published in 2013, which complied with the authors’ inclusion criteria, described an “indication” of an increased risk of MI in the early stages of therapy with the NRTI, abacavir (RR 1.92, 95% CI 1.51–242), as well as with protease inhibitors (RR 2.13, 95% CI 1.06–4.28) (120). The authors conceded, however, that “our findings of increased cardiovascular risk from abacavir and protease inhibitors were in contrast to four published meta-analyses based on secondary analyses of randomized controlled trials, which found no increased risk from cardiovascular disease” (120). In the case of abacavir, Llibre and Hill in their recent review have proposed that the currently available evidence from clinical studies, together with lack of mechanistic data, is not supportive of the involvement of this agent in “causing a short/middle (but not cumulative) increase in the risk of acute MI or cardiovascular events” (121).

### Profibrotic Mechanisms of Myocardial Dysfunction

Myocardial fibrosis, of which TGF-β1 is a key mediator (122), is widely recognized as “a significant global health problem associated with nearly all forms of heart disease” (123) and has recently been identified as being a probable significant contributor to the excess incidence of HIV-related CV events (94, 124–126). In this context, Thiara et al., using magnetic resonance imaging (MRI), investigated the possible association of intramyocardial lipid accumulation and fibrosis with HIV-related myocardial dysfunction in a total of 95 adult patients, the majority of whom (93%) were receiving HAART (124). These authors observed that relative to matched healthy controls, systolic function was significantly impaired in HIV-infected participants and associated with significantly increased intramyocardial lipid accumulation and fibrosis (124). The following positive correlations were also noted: (i) intramyocardial lipid levels with duration of HAART and visceral adiposity, although the number of participants was too small to identify possible associations with individual antiretroviral agents and (ii) myocardial dysfunction with systemic biomarkers of inflammation, specifically lipopolysaccharide (LPS)-binding protein and monocyte chemoattractant protein 1. Taken together these findings are seemingly consistent with the involvement of both HAART and microbial translocation in the pathogenesis of HIV-related abnormal cardiac function (124). The authors concluded that “increased subclinical cardiac dysfunction is associated with cardiac steatosis and fibrosis in HIV-infected adults” (124).

The findings of the study reported by Thiara et al. (124) were confirmed and extended in two subsequent studies, which were also based on advanced MRI procedures, to which 22 and 92 HAART-treated, asymptomatic HIV-infected participants were recruited (125, 126). The authors documented the existence of subclinical myocardial inflammation, edema and fibrosis, which were associated with alterations in myocardial structure and function (125, 126). In one of these studies, myocardial fibrosis, which was detected “predominantly at the subepicardium of the midventricular and basal inferolateral wall,” was present in 82.1 and 27.3% of HIV-infected and healthy control participants, respectively (*p* < 0.001). The authors of both studies proposed chronic systemic inflammation involving the myocardium and pericardium to be the cause of myocardial fibrosis and associated CV morbidity and mortality (125, 126). However, conclusive support for this contention is dependent on the outcome of larger studies of this type which should also include extensive cardiac and inflammatory biomarker profiles (94), including TGF-β1 in particular.

## Renal Disease and Its Relationship to HAART-Mediated Toxicity and Inflammation-Associated Fibrosis in HIV Infection

From the early stages of the HIV pandemic, HIV-infected persons were recognized to have an increased risk of renal disease. Proteinuria and/or raised creatinine have also been associated with an increased risk of progression to AIDS and death in both the pre-HAART and HAART era (127). While it is possible that kidney disease may modulate HIV disease progression, these markers may merely be indicative of greater comorbidity (127). For instance, both acute kidney injury (AKI) and chronic kidney disease (CKD) are associated with CVD, which, as mentioned above, has become a leading cause of morbidity and mortality in HIV-infected populations (128).

Acute kidney injury can be attributed to prerenal, renal, and postrenal factors (129). The most common causes in the HAART era are intrinsic to the kidney, namely ischemic acute tubular necrosis, often in the context of an infection, or nephropathy secondary to nephrotoxic medication (130, 131). A study of a nationally representative database in the United States noted a twofold increase in the incidence of AKI requiring dialysis among hospitalized HIV-infected adults between 2002 and 2010, partially attributed to an increase in the prevalence of chronic comorbidities known to be risk factors for development of AKI (131).

Chronic kidney disease has also emerged as a major comorbid condition with HIV-infected persons estimated to have an almost fourfold increased risk. In addition to traditional risk factors, such as older age, hypertension, smoking and diabetes mellitus, late stage disease (i.e., low CD4 count and high HIV viral load) and coinfection with HCV have been identified as significant risk factors (132). Apart from glomerulonephritis secondary to hepatitis B virus (HBV) and HCV infection, HIV-related disorders, including HIV-associated nephropathy (HIVAN), HIV immune complex kidney disease (HIVICK), and less commonly, HIV-associated thrombotic microangiopathy, have emerged as important etiologies of CKD. CKD has a typical phenotype, characterized by a progressive reduction in nephrons associated with increased fibrosis and interstitial scarring, regardless of etiology (133). Many glomerular, tubular, and inflammatory processes involved in the development of this phenotype are thought to be mediated by TGF-β (134). Importantly, TGF-β increases expression of trophic and profibrotic factors, such as angiotensin II and connective tissue growth factor, as well as other pathological mediators, such as TNF-α and interleukins, that promote the formation of myofibroblasts (133, 135).

### HIV-Associated Nephropathy

HIV-associated nephropathy is the pathognomonic form of HIV-related renal disease and is one of the leading causes of end-stage disease. This is especially true in patients of African descent due to high frequencies of apolipoprotein L1 risk alleles in this population (136, 137). HIVAN is characterized by a collapsing form of focal segmental glomerulosclerosis (FSGS), formation of tubular microcysts, inflammation in the tubulointerstitial space, and fibrosis (136, 138). FSGS has been proposed to have an inflammatory character similar to that of atherosclerosis, marked by the accumulation of cholesterol and cholesterol esters, activation of monocytes, production of lipid-laden macrophages, and expansion of contractile cells and matrix, leading to fibrosis (139). The pathogenesis of HIVAN is not fully understood, but seems to be triggered by direct viral infection and viral protein R-induced apoptosis of renal tubular epithelial cells, Nef-induced podocyte dysfunction, and upregulation of proinflammatory mediators, especially those induced by NF-κB (136).

The fibrogenic action of TGF-β, centered on its ability to induce extracellular matrix deposition in the glomeruli and tubular interstitium, is also believed to play an important role in the development of the pathological tissue fibrosis observed in HIVAN (140). Glomerular and tubulointerstitial expression of TGF-β has been found to be increased in experimental models and histological samples of patients with glomerulopathies that feature pathological extracellular matrix accumulation (141, 142). Recently, higher plasma levels of TGF-β have also been found to be associated with lower estimated glomerular filtration rate (143). In HIVAN, the mechanism underpinning the increase in TGF-β is proposed to be the HIV gene product, Tat protein, which has been shown to stimulate production of TGF-β by macrophages (144) and mesangial cells (140). TGF-β further stimulates HIV replication (145), thereby increasing Tat and inducing a vicious cycle of virus replication, TGF-β expression, and matrix deposition (140). Together with reduced expression of matrix proteins and overexpression of matrix remodeling enzymes, such as MMP-9, TGF-β is proposed to regulate the sclerosing and collapsing features of HIVAN (137). This is supported by the observation that the therapeutic effects of angiotensin II blockers in HIVAN correlate with decreased TGF-β-induced renal fibrosis (146).

HIV-associated nephropathy is principally seen in patients with advanced disease and has become less common in the HAART era (137). In contrast, diabetic nephropathy and arterionephrosclerosis have become more common, presumably secondary to chronic inflammation and dysmetabolism that persists in patients on virally suppressive treatment (137). Both pathologies have been associated with activation of TGF-β1 signaling with elevated levels of TGF-β1, mediated through increased levels of renin and angiotensin II (147, 148).

### HAART-Related Renal Toxicity

With more people on long-term treatment, HAART-induced nephrotoxicity is becoming an increasing problem with TDF-induced proximal tubular dysfunction and protease inhibitor-associated crystalluria the most common. TDF-associated nephrotoxicity mostly manifests as AKI, CKD, or proximal tubular injury secondary to mitochondrial damage caused by inhibition of polymerase γ (149). The protease inhibitor, indinavir, and to a lesser extent atazanavir, is poorly soluble in urine and can potentially cause crystalluria with subsequent interstitial nephritis and, rarely, parenchymal fibrosis (150). Although some medications, specifically those employed as immunosuppressants, have been associated with diffuse fibrosis and sclerosis secondary to increased expression of TGF-β (151), direct links between indinavir-mediated fibrosis and TGF-β1 have not been evaluated.

## Liver Disease and Its Relationship to Inflammation-Associated Fibrosis in HIV Infection

Liver disease is also increasingly being recognized as one of the leading causes of non-AIDS-related mortality and morbidity among HIV-infected individuals (73, 152). During HIV infection, a large number of cytokines and chemokines, including TGF-β1, as well as platelet-derived growth factor and endothelin I that promote the inflammatory response, fibrosis, contraction and mitosis, are released from Kupffer cells, lymphocytes, liver sinusoidal endothelial cells, platelets, and hepatic stellate cells (HSCs) (153, 154). HIV has been shown to modulate the activation and functioning of HSCs which are the main effector cells responsible for the fibrotic process (155–157). HSCs are non-parenchymal cells which, in their quiescent state, are responsible for the storage and metabolism of vitamin A. Following liver injury and/or viral infection, HSCs are transformed into proliferative, fibrogenic, proinflammatory, and contractile myofibroblasts that actively produce extracellular matrix components such as type I collagen (158). Accumulation of these proteins due to dysregulated release of the MMPs by HSCs leads to replacement of the liver parenchyma by scar tissue, resulting in liver fibrosis and its associated complications (95). Activated HSCs, in turn, perpetuate their own activation through several autocrine loops, including the secretion of additional TGF-β and upregulation of its receptors (159).

A number of other liver-related comorbidities with a profibrotic component are associated with HIV-infection, particularly in the setting of coinfection with HBV or HCV (5–30 and 15–40%, respectively, depending on the geographical region) (160–162). In this context, it is noteworthy that expression of TGF-β1 has been found to be significantly increased in both the serum and in the liver of individuals with HIV/HCV coinfection (163, 164). Other scenarios which predispose to and/or exacerbate HIV-associated chronic liver disease and hepatic fibrosis are alcohol/drug abuse, and the use of hepatotoxic traditional/herbal remedies (165–167), as well as metabolic abnormalities (insulin resistance, diabetes mellitus, and lipodystrophy syndrome) (166, 168).

Increased bacterial translocation, immune activation, and the presence of soluble proteins that alter the hepatic cytokine environment, producing a proinflammatory milieu, are also linked to direct and indirect profibrotic effects of HIV (152, 169). Although unproven, persistently elevated levels of circulating TGF-β1, consequent to chronic immune activation, may also contribute to the pathogenesis of HIV-related fibrotic disorders of the liver. In this context, it is noteworthy that microbial translocation promotes LPS entry into the peripheral circulation leading to increased TLR-4 signaling in Kupffer cells, with resultant generation of ROS and release of proinflammatory and profibrotic cytokines. This mechanism, in turn, exacerbates liver damage, compromising the ability of the liver to metabolize circulating LPS and promoting progression to liver fibrosis (170). Hepatic dysfunction, in turn, results in sustained elevations in the levels of circulating LPS due to reduced clearance by Kupffer cells (170).

Importantly, the introduction of HAART has also been associated with the emergence of a number of liver conditions including acute toxic hepatitis, steatosis, non-alcoholic steatohepatitis, non-alcoholic fatty liver disease and non-cirrhotic portal hypertension (157, 165, 171). However, the involvement of TGF-β1, if any, in the pathogenesis of these disorders remains to be established. Notwithstanding potential hepatotoxicity which has recently been extensively reviewed elsewhere (167), the short- and mid-term effects of HAART on the liver are, however, mostly considered to be beneficial, especially in HBV or HCV coinfected individuals, and are considered to outweigh the potential risks of long-term toxicity (172, 173).

## Pulmonary Disease and Its Relationship to Inflammation-Associated Fibrosis in HIV Infection

With the notable exception of lung cancer, chronic obstructive lung disease (COPD) and pulmonary hypertension are the most common non-AIDS-defining pulmonary NCDs, occurring at higher frequencies than in the general population (174). Although compounded by a high prevalence of cigarette smoking among those living with the virus (175, 176), HIV/AIDS is now recognized as an independent risk factor for the development of COPD (174), a condition which has been identified as an emerging and serious threat to health in the HAART era (177, 178). With respect to the possible involvement of fibrotic mechanisms in disease pathogenesis, TGF-β1, *via* its profibrotic role in airway remodeling, has been reported to contribute to airflow limitation and disease severity in HIV-uninfected persons with COPD (179–181). Not surprisingly, albeit much less extensively characterized than in the clinical settings of HIV-related profibrotic disorders of CV, hepatic and renal origin, HIV infection has recently been reported to be associated with fibrosis-like changes in the lungs of a significant percentage (29%) of patients, correlating with viral load, but not with either HAART or CD4 count (182). In this context, the findings of an earlier study which reported excessive production of TGF-β by alveolar macrophages from HIV-infected persons are noteworthy (183). Nonetheless, the possible involvement of TGF-β1 in the pathogenesis of pulmonary fibrosis and COPD in the setting of HIV infection remains to be established.

## TGF-β-Targeted Therapeutic Strategies

Transforming growth factor beta has been described by Meng et al. in their recent review as being “the master regulator of fibrosis” (184). In their review, the authors have clearly described and depicted TGF-β-receptor-activated intracellular signaling mechanisms, identifying a range of potential therapeutic targets applicable to various inflammatory disorders, possibly including HIV/AIDS (184) a consideration of which is beyond the scope of this review. Of the many agents currently undergoing clinical evaluation in various fibrotic disorders (185), the anti-inflammatory/antioxidative drug, pirfenidone, and the α_v_β6 integrin-targeted monoclonal antibody, STX-100X, have shown promise in the treatment of idiopathic pulmonary fibrosis (186, 187). With respect to pirfenidone, Estes et al. have reported that administration of this agent to SIV-infected rhesus macaques resulted in protection against lymphoid tissue, paracortical T cell zone, fibrosis (188). This, in turn, was associated with significant increases in the numbers of CD4^+^ T cells in peripheral blood and lymphoid tissues (188). The authors advocate antifibrotic drug/HAART combination therapy as a potential strategy to support recovery of immune function (188). Enthusiasm for this approach must, however, be countered by an awareness of the key protective biological activities of this cytokine. Alternative, and potentially safer strategies include the early implementation of efficacious antiretroviral therapy, reversal of increased intestinal permeability with probiotics, and the development of anti-inflammatory agents which effectively target intracellular signaling pathways common to those TLRs which are activated by bacterial LPS, bacterial DNA, and viral single-stranded RNA (TLRs-4, -7, -8, -9), as well as combinations of these.

## Conclusion

Sustained elevations in circulating TGF-β1 are believed to contribute not only to immunosuppression and progression to AIDS, but also to residual immunosuppression in virally suppressed persons. In addition, the profibrotic activity of the cytokine, which also contributes to immunosuppression, may be linked to the pathogenesis of non-AIDS-defining cardiovascular, hepatic, renal, pulmonary and other disorders. Consequently, TGF-β1 (and possibly the other isoforms of this cytokine), represents an alternative, largely unexplored, therapeutic target in HIV infection which is dependent on the development of safe and efficacious strategies to counter the harmful activities of this cytokine.

## Author Contributions

All the authors contributed significantly to the planning and compilation of the manuscript.

## Conflict of Interest Statement

The authors declare that the research was conducted in the absence of any commercial or financial relationships that could be construed as a potential conflict of interest.
